# Effect of Temperature and Branched Crosslinkers on Supported Graphene Oxide Pervaporation Membranes for Ethanol Dehydration

**DOI:** 10.3390/nano10081571

**Published:** 2020-08-10

**Authors:** Azeem Bo Strunck, Anil Suri, Vittorio Boffa

**Affiliations:** Center for Membrane Technology, Department of Chemistry and Bioscience, Aalborg University, Fredrik Bajers Vej 7H, 9220 Aalborg Øst, Denmark; azeembs@bio.aau.dk (A.B.S.); vb@bio.aau.dk (V.B.)

**Keywords:** pervaporation, membrane, separation, biofuel, azeotrope, graphene oxide

## Abstract

We describe the performance of graphene oxide (GO) membranes stabilized by crosslinkers and supported on polyethersulfone films in the dehydration of ethanol in a continuous cross-flow pervaporation set-up. We used two crosslinker species with branched structures (humic acid-like substances derived from urban waste and a synthetic hyperbranched polyol). The supported crosslinked GO films were prepared by rod coating on a polyethersulfone ultrafiltration membrane. Pervaporation experiments were carried out at temperatures of 40, 50, 60 and 70 °C. When the feed comprised pure water and ethanol, a much higher flux of water than ethanol was observed at all temperatures through GO films stabilized by the two crosslinkers (humic acid, GO-HAL, and the synthetic hyperbranched polyol, GO-HBPO), indicating the separation ability of these crosslinked membranes. For feed mixtures of water and ethanol, the GO-HAL and GO-HBPO membranes showed good separation performances by producing permeates with a significantly higher water content than the feed at all temperatures.

## 1. Introduction

Biofuels, including biodiesel and bioethanol, are being regarded as promising environment-friendly alternatives to fossil fuels for future energy requirements, as their use will lead to lower CO_2_ emissions [1,2,3,4]. Bioethanol, produced by the fermentation of low-cost biomass, has a high water content and must be dehydrated [4,5]. However, ethanol forms an azeotrope with water at 1 atm and 78.2 °C at a composition of 89.4 mol% (or 95.6 wt%) of ethanol, which hinders dehydration by distillation. Therefore, alternative separation processes such as azeotropic distillation, vacuum distillation and desiccation are being considered [6,7]. These processes are energy-intensive, and low-cost alternatives are actively being sought. To this end, membrane separation processes like membrane distillation, vapor permeation and pervaporation are being explored [5,8,9].

In pervaporation, the property of the membrane material plays an important role in the separation process, owing to which the separation is not limited by the difference in the volatilities of the components to be separated [9]. Membranes made from a variety of materials have been studied for the dehydration of ethanol by pervaporation, including polymers such as polydimethylsiloxane [10], polydimethylsiloxane-polystyrene [11] and polyvinyl alcohol-polyacrylamide [12] interpenetrating polymer networks, inorganic materials such as zeolites [13] and hybrids/composites such as polyvinyl alcohol–sodium alginate [14] and polyvinyl alcohol–zeolite [15], although most commercially available membranes for ethanol dehydration today are based on polyvinyl alcohol, which was also the first to be industrially used for this purpose in the early 1980s [16].

Recently, the potential of graphene as a membrane material has drawn considerable research attention after it was reported that graphene sheets are impervious to gases such as air, argon and helium [17], and graphene oxide (GO) membranes permit the virtually unhindered diffusion of water while blocking other gases and vapors [18]. GO has hydrophobic and hydrophilic domains, and it has been proposed that water molecules are transported across the GO membrane by gliding without resistance over the hydrophobic domains and diffusing through the network of pores between the stacked GO sheets [18,19].

GO also has the advantage of being dispersible in water, which makes possible the easy synthesis of GO membranes by low-cost, scalable solution processing. However, this also leads to problems of stability. This can be overcome by the partial reduction of GO. However, reduction leads to the restoration of close-packing and a decrease in the spacing between the reduced GO sheets [20,21], resulting in a decline in flux. In addition, the room-temperature chemical structure of GO is metastable and changes continuously with changes in temperature and exposure to water as a result of partial reduction [22,23]. This, in turn, can change the spacing between the GO layers, resulting in fluctuations in throughput over time. For this reason, a considerable amount of study is required to understand the long-term behavior and reliability of these membranes before they can be regarded as a viable option for large-scale commercial application. To ensure high flux, approaches such as the incorporation of water channel proteins in GO membranes [24], the use of hybrids of GO and hydrophilic polymers [25] and the use of spacers such as carbon nanotubes [26] and carbon nanodots [27] between the graphene sheets have been investigated.

Chemical crosslinking of GO sheets is another frequently reported method for the stabilization of GO membranes. GO sheets have oxygen-containing functional groups such as carboxylic acids, alcohols, epoxides and carbonyls. Molecules such as diols, dicarboxylic acids [28], diamines [29,30] and dialdehydes [31] can form covalent bonds with these functional groups, thereby tethering both adjacent as well as parallel GO sheets. When the crosslinker molecules are normal to the GO sheets, they can act as spacers between them and hence can be used to regulate the flux through the membrane [29]. Further, many techniques are being studied for the scalable production of both free-standing as well as supported GO membranes, including filtration [28,29,30], drop casting [32], layer-by-layer deposition [33,34,35], dip coating [36,37], vacuum suction [38], spray coating [39,40] and rod coating [39,41]. We have previously studied the stability of GO membranes obtained by crosslinking GO by molecules with branched structures, as well as the potential of the membranes for the dehydration of ethanol–water mixtures beyond the azeotropic limit using a vapor permeation set-up [41]. Two crosslinkers were studied: humic acid-like substances (HAL) obtained from urban wastes, and a synthetic hyperbranched polyol (HBPO), namely, hyperbranched bis-MPA polyester-16-hydroxyl, generation 2. The supported GO membranes were fabricated by Meyer rod coating on commercial polyethersulfone films, which is amenable to scaling up to an industrial scale. We had also assessed the stability of free-standing crosslinked GO membranes under prolonged immersion in water. However, the membranes have not been tested under realistic cross-flow conditions yet. Moreover, the vapor permeation experiments carried out so far on the supported membranes involved only vapor in contact with the membranes on the feed side, although they demonstrated the potential of our membranes for the dehydration of ethanol/water mixtures beyond the azeotropic limit. Herein, we study the suitability and performance of the supported membranes in a continuous cross-flow pervaporation set-up where the liquid feed is in direct contact with the membranes. For comparison, we also studied supported membranes of pure GO without crosslinking and GO stabilized by a linear crosslinker, poly(ethylene glycol).

## 2. Materials and Methods

### 2.1. Materials

The hyperbranched polyol (HBPO), viz. hyperbranched bis-MPA polyester-16-hydroxyl, generation 2, molecular weight 1750 Da (97%), sodium nitrate (>99% purity), potassium permanganate (>99% purity) and hydrogen peroxide (30% w/w in water) were obtained from Aldrich. Poly(ethylene glycol) with molecular weight 2000 (PEG2000) was obtained from Fluka. Absolute ethanol (≤0.1% water) was obtained from VWR. Polyethersulfone membrane (molecular weight cutoff 100,000 Da) was supplied by Synder Filtration (Vacaville, CA, USA). The humic acid-like crosslinker (HAL) was isolated from a commercial green compost, produced by ACEA Pinerolese Industriale S.p.A. in its waste treatment plant in Pinerolo (Italy) by aging the trimming residues from public parks and home gardening for 180 days. The detailed procedure for HAL isolation is described elsewhere [32].

### 2.2. Preparation of GO and Fabrication of Supported GO Membranes

Graphene oxide (GO) dispersions were prepared from natural graphite (Graphit Kropfmühl GmbH, Hauzenberg, Germany) as described elsewhere [33]. GO films supported on flat-sheet polyethersulfone membrane (hereafter referred to as “supported GO membranes” or simply “GO membranes”) were prepared as described by us earlier, by Meyer rod-coating (TQC B.V., Capelle aan den IJssel, The Netherlands; application length 320 mm, film thickness 50 µm) of GO aqueous suspension on polyethersulfone membranes [41]. Table 1 summarizes the properties of the four types of supported GO membranes.

Using Meyer rod coating, it was possible to prepare large-area supported GO membranes of a width of around 250 mm (limited by the application length of the Meyer rod). In principle, the membranes can be prepared to any desired length, but we used polyethersulfone supports cut approximately as squares for rod coating by hand. We have previously found that the thickness of the dry GO layer on the polyethersulfone support is around 20–25 µm [41]. The large-area membranes were cut in 75 mm × 58 mm samples, which were used for cross-flow pervaporation experiments.

### 2.3. Cross-Flow Pervaporation Experiments

**Figure 1:**
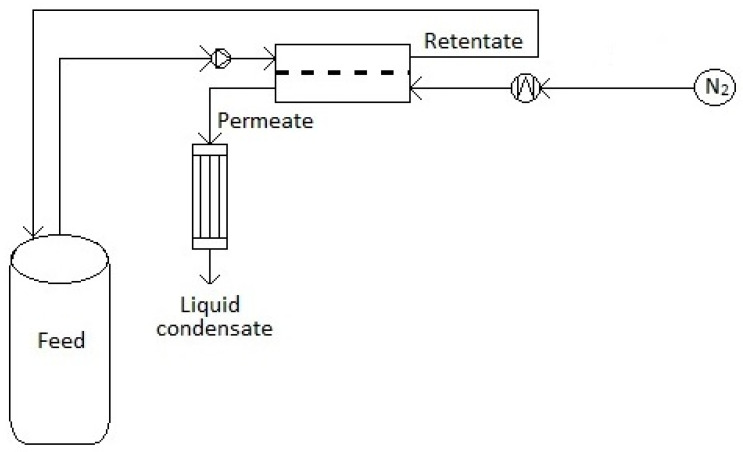
Schematic depiction of the cross-flow membrane separation apparatus used in this study.

The set-up for the continuous flow membrane separation process is similar to that used by Tang and co-workers [42] and is shown schematically in Figure 1. The feed (pure water, absolute ethanol or ethanol/water mixture of known composition, taken in a starting quantity of ~1 kg) is maintained at a constant temperature in a still and pumped at a flow rate of 1.26 × 10^−5^ m^3^/s to a Pervatech CF042-FO cell, also maintained at a constant temperature in a water bath. Hybrid distillation/pervaporation processes have been suggested to optimize the cost of separation, with the amount to be separated by the membrane ideally being as small as possible [43]. Therefore, two similar compositions of water/ethanol mixtures close to the azeotropic composition were studied as feed, one with approximately 88 wt% ethanol and the second with 92 wt%. The closeness of the two feed compositions studied also makes it easier to verify the reproducibility of our data. The exact composition of the feed mixture was determined by coulometric Karl Fischer titration (Mettler Toledo C20 coulometric KF, Columbus, OH, USA) before the run. However, for the sake of convenience, they are labeled as 88% and 92%. The runs were carried out at four different temperatures: 40, 50, 60 and 70 °C. The retentate was recycled to the feed still. The permeate was swept out by a continuous flow (3 L min^−1^) of nitrogen gas and condensed in two cold traps (maintained at a temperature of −20 °C) in a sequence to minimize the loss of vapor with the vented nitrogen. The weight of the permeate collected after a known duration was recorded. The duration of each run was between 1 and 2 h. The weight of the permeate obtained at the end of a run ranged between 5–20 g, i.e., less than 2% of the initial feed weight, which would result in negligible changes in the composition of the feed during the course of a run. The percentage of water in the permeate was also determined by Karl Fischer titration.

The membrane performance parameters, i.e., the permeances of the components, the membrane selectivity and separation factors, are calculated as defined by Baker et al. [44].

The permeance, *P_i_*, of each component is defined as:

*P_i_* = *J_i_*/(Δ*p_i_*) (1)

where *J_i_* is the molar flux, and Δ*p_i_* is the partial pressure (driving force), of component *i*.

The membrane selectivity, *α*, is calculated as the ratio of the permeance of water to that of ethanol:

*α* = *P_water_*/*P_ethanol_* (2)

The separation factor, *β*, is determined as:

(3) $$\beta =\frac{y_{water}\times x_{ethanol}}{x_{water}\times y_{ethanol}}$$

where *x* and *y* refer to the mole fractions of the components in the feed and permeate, respectively [45,46].

## 3. Results and Discussion

### 3.1. Pure Component Feed

**Figure 2:**
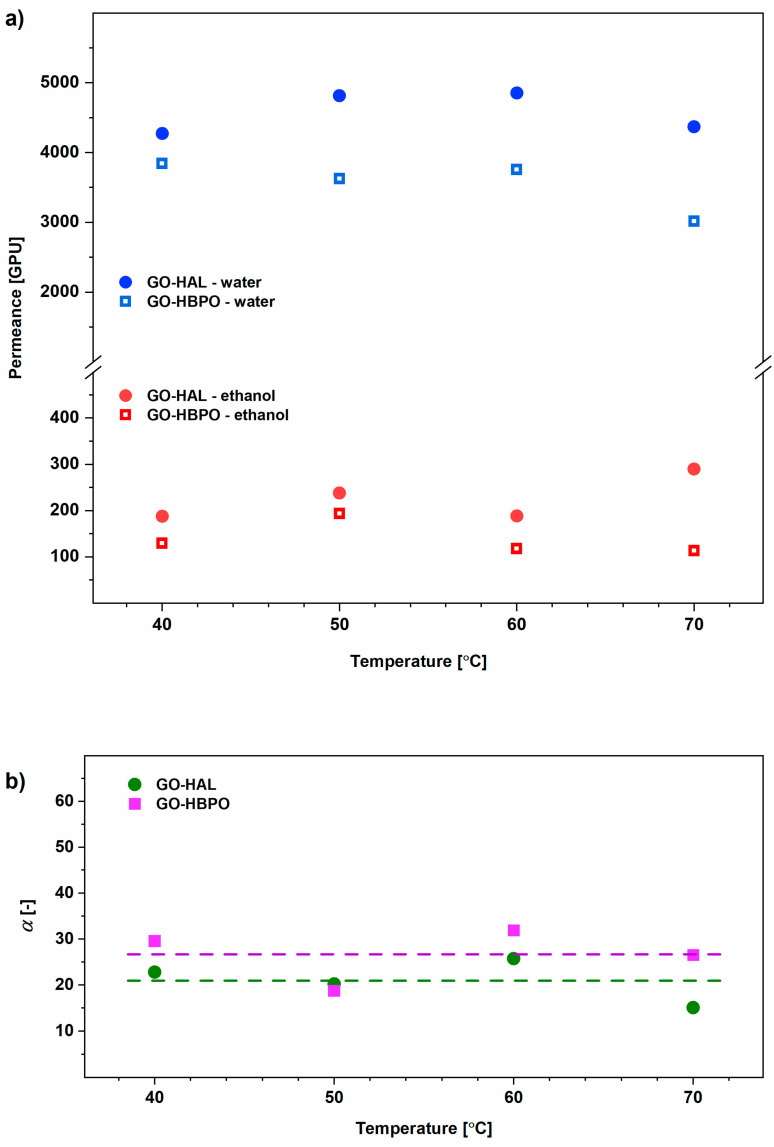
(**a**) Permeance of pure water (blue symbols) and absolute ethanol (red symbols) through GO-HAL (humic acid-like crosslinker) and GO-HBPO (hyperbranched polyol) membranes, and (**b**) ideal water/ethanol selectivity of the two membranes as a function of the feed temperature (°C); dotted lines indicate the average of the selectivities for the two membranes. The permeance values have been expressed in Gas Permeation Units (GPUs), where 1 GPU = 3.35 × 10^−10^ mol·(m^2^·s·Pa)^−1^ [47].

Figure 2a shows the permeance of pure water and absolute ethanol for the GO-HAL and GO-HBPO membranes. When single components are tested, the permeance can be simply calculated by dividing the molar flux of the permeate by the vapor pressure of the component at the feed side of the membrane [45]. GO-HAL and GO-HBPO showed a much higher permeance of pure water than absolute ethanol at all temperatures, underscoring the separation efficiency of the membranes. Over three measurements, the maximum standard deviation is 6.6% for the permeance of water and 13.6% for that of ethanol. The supported GO-HAL membrane shows a water permeance that is one order of magnitude larger than what we previously measured for the corresponding unsupported, and therefore thicker, GO-HAL films [32]. Moreover, the permeances of pure water and absolute ethanol through the GO-HBPO membrane are lower than those for GO-HAL. This is explained in terms of the availability of multiple free –OH groups on the crosslinker molecules in GO-HBPO, which bind with the water and ethanol molecules via hydrogen bonding, hindering their diffusion across the membrane; such binding sites are less available in the HAL, and hence, the water and ethanol molecules are transported across the membrane with less impediment. Figure 2b depicts the ideal selectivities of the two membranes, which are here calculated as the ratio of the permeance of water to that of ethanol at each temperature. These data show that both membranes have ideal selectivities in line with those previously observed in vapor permeation experiments [41], and they are both stable under the cross-flow filtration conditions for the duration of the study, i.e., one week.

A membrane prepared by coating pristine GO on polyethersulfone support suffered considerable leakage, with large amounts of water and ethanol accumulating in the traps in a very short period of time, owing to which the experiment had to be stopped. This is attributable to the non-selective nature of the polyethersulfone support and the poor stability of the GO membrane. In our previous study, we had also reported the vapor permeation performance of GO crosslinked by linear poly(ethylene glycol) [41]. Although this membrane had shown stability as well as separation performance comparable to the GO-HAL and GO-HBPO in our earlier study, it unexpectedly failed. This membrane also showed considerable leakage and was unsuitable for cross-flow pervaporation experiments. This underscores the stability conferred by the branched-structure HAL and HBPO crosslinkers, making them amenable for cross-flow pervaporation.

### 3.2. Ethanol–Water Mixtures

**Figure 3:**
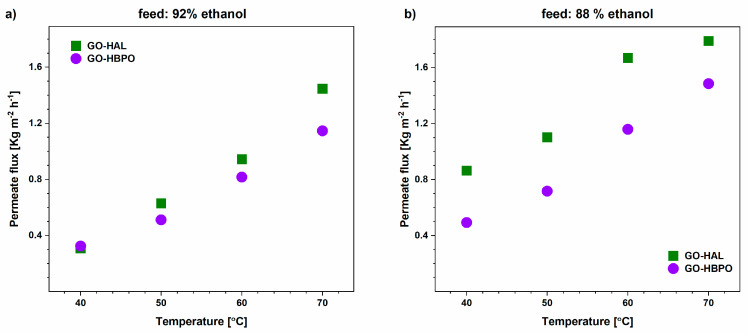
Total flux through GO-HAL and GO-HBPO as a function of the feed temperature for ethanol/water mixtures containing (**a**) 92% ethanol and (**b**) 88% ethanol.

The values of the total mass flux through the membranes are shown in Figure 3. Our values (0.25–1.8 kg m^−2^ h^−1^) are significantly higher than those reported, for instance, by Hua et al. [31] (0.2–0.4 kg m^−2^ h^−1^) and Cheng et al. [48] (0.1–0.45 kg m^−2^ h^−1^). In fact, only two of the 16 values of flux in Figure 3 are less than 0.45 kg m^−2^ h^−1^. Recently, in an approach very similar to ours [41], Shin and co-workers reported the preparation of GO membranes supported on polyethersulfone films by a casting method and their performance in ethanol dehydration [49]. For a 90 wt% ethanol feed, they achieved a total flux of less than 0.3 kg m^−2^ h^−1^ and a maximum flux of 1 kg m^−2^ h^−1^ for a 5% ethanol feed at an operating temperature of 90 °C, which is lower than the flux through our crosslinked GO-HAL membranes over the entire range of 30–70 °C and the GO-HBPO membranes at 60 and 70 °C. Moreover, both GO-HAL and GO-HBPO show higher permeation rates when tested with a feed with 88% than that with 92%, ethanol, suggesting a higher permeance of water over ethanol in binary mixtures too.

**Figure 4:**
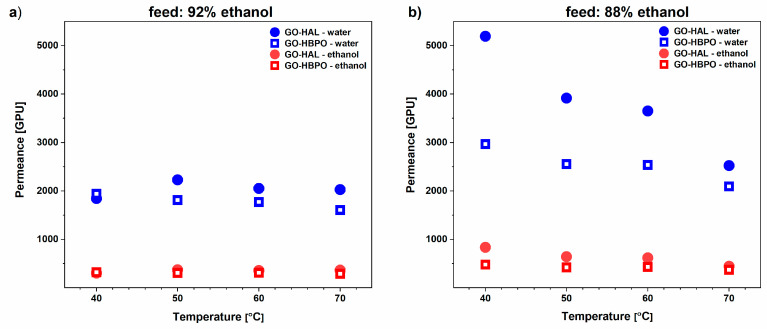
Permeance of water (blue symbols) and ethanol (red symbols) components at four operating temperatures for GO-HAL and GO-HBPO membranes for ethanol/water feeds with (**a**) 92% and (**b**) 88% ethanol. The permeance values have been expressed in Gas Permeation Units (GPUs), where 1 GPU = 3.35 × 10^−10^·mol (m^2^·s·Pa) ^−1^ [47].

The permeances of the water and ethanol fractions, i.e., the molar flux of the component normalized by its partial pressure (driving force) for the two membranes, are shown in Figure 4. The partial pressures are estimated by determining the activity coefficients for water and ethanol by the group contribution method [50]. For both types of membranes, the permeance of water is significantly higher than that of ethanol for both feed compositions and at all temperatures, confirming good separation. Significantly, the permeance of the water in the feed mixtures is much lower than that observed for pure water (Figure 2), while the permeance of the ethanol component is much higher than that of absolute ethanol. For instance, for GO-HBPO, the permeances of the water component of the 88% and 92% feed mixtures (Figure 4) are only around 70% and 50%, respectively, of the permeance of pure water (Figure 2) at the same temperature. However, the permeance of the ethanol component for the 88% and 92% feed mixtures is more than three times and two times, respectively, than that of absolute ethanol at the same temperature. This can be explained as the combined effect of two phenomena. First, the increase in interlayer spacing in the GO membrane owing to the incorporation of water [51] and ethanol [52] between the layers increases the permeance through the membrane. Second, as the diffusion pathways of ethanol and water partially overlap, an increase in the amount of the relatively more volatile ethanol intercalated in the GO membrane at higher temperatures occludes the diffusion pathways and reduces the permeance of water. Furthermore, in general, the permeance of water decreases significantly with a relatively small increase in the proportion of ethanol in the feed. For instance, for the GO-HAL membrane, the water permeance at 40 °C for the 88% feed is almost three times that for the 92% feed (Figure 4).

**Figure 5:**
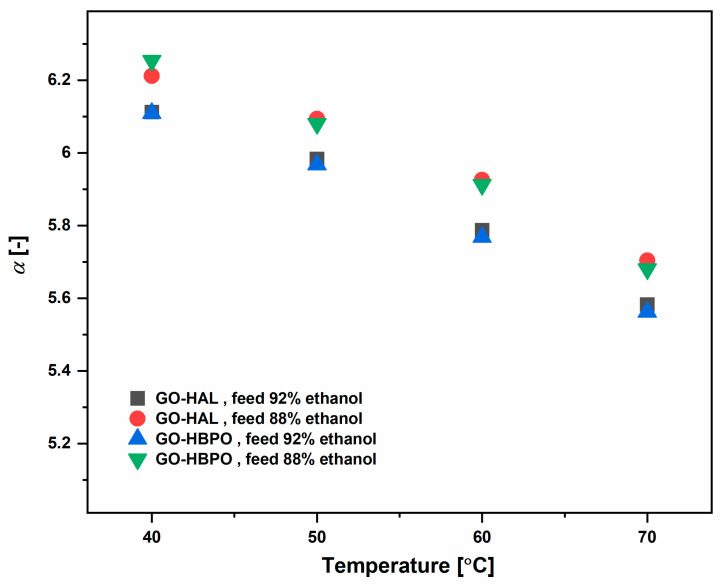
Selectivities of the two types of crosslinked membranes for both feed compositions at the four operating temperatures.

The selectivities for both feed compositions and at all four operating temperatures of both types of membranes are shown in Figure 5. The selectivities follow an identical decreasing trend with increasing temperature and corroborate the reproducibility of our results. For testing the veracity of our results, the GO-HAL experiments were carried out using different membranes for the two feed mixtures, while the same GO-HBPO membrane was used for both the 92% and 88% feeds.

**Figure 6:**
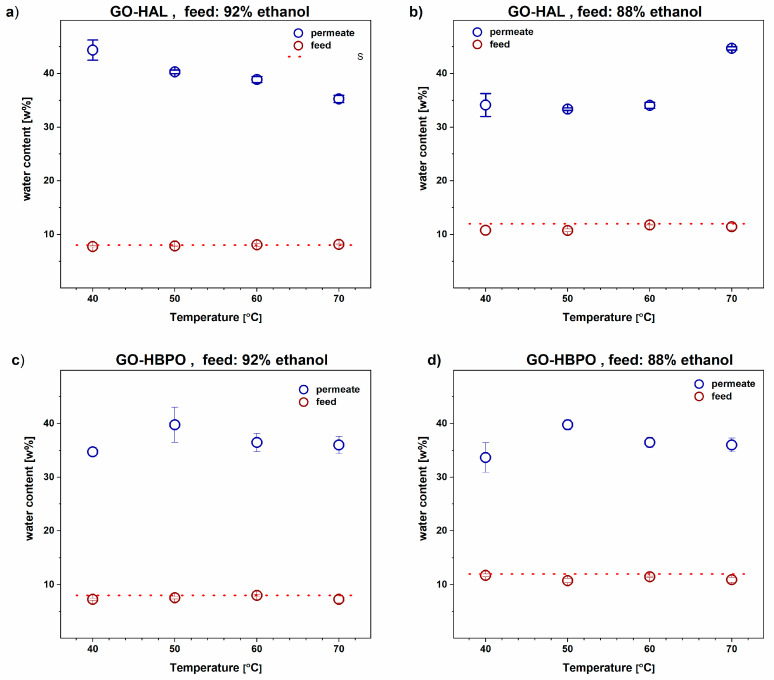
Wt% of water in feed and permeate for the membrane and feed combinations of (**a**) GO-HAL, 92%, (**b**) GO-HAL, 88%, (**c**) GO-HBPO, 92%, and (**d**) GO-HBPO, 88%. Dotted lines indicate the theoretical water content in the feed.

For ethanol/water mixtures, the wt% values of water in the feed and in the permeate are shown in Figure 6. As can be seen, the proportion of water in the permeate is always significantly higher than in the feed, implying that the retentate has been enriched in ethanol. For GO-HAL and the 92% feed (Figure 6a), the permeate water content steadily decreases with feed temperature, from around 45% at 40 °C to around 35% at 70 °C. This straightforward trend is owing to the fact that, at higher temperatures, the vapor pressure of ethanol is higher, creating a higher driving force for its diffusion through the membrane. When the water content in the feed increases slightly (88% feed), the permeate water content remains nearly constant as the feed temperature increases from 40 to 60 °C, but increases noticeably at 70 °C (Figure 6b). For GO-HBPO, for both feed compositions (Figure 6c,d), the permeate water content first increases from around 35% at 40 °C to a maximum of 40% at 50 °C, before declining to around 36% at 70 °C. Once again, these trends are the result of the two factors influencing the transport through the membrane as described above: the intercalation of water and ethanol molecules from the feed in the membrane increases the spacing between the GO layers, which enhances the flux through the membrane, but a higher proportion of ethanol in the feed vapor blocks the diffusion pathways of the water molecules. These observations suggest that the membrane separation performance is very sensitive to the feed conditions. In addition, the permeates contain large proportions of ethanol. We have shown that incorporation of crosslinkers with branched structures like HAL in the GO membrane can induce a significant amount of disorder, which results in a much higher flux than in pristine GO, although it leads to a decline in the water/ethanol separation factor [32].

**Figure 7:**
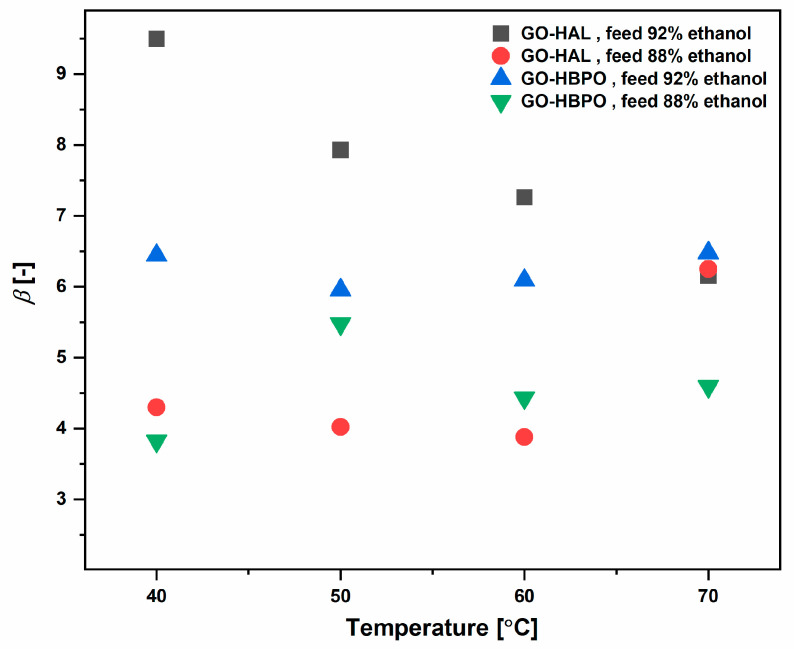
Separation factors for the pervaporation runs.

The separation factors of the crosslinked GO membranes for various conditions are shown in Figure 7. Our separation factors are modest compared with those reported by others, such as Hua et al. for aldehyde-crosslinked GO membranes [31] and Castro-Muñoz et al. for PVA membranes incorporated with GO [53]. This is owing to the relatively high amount of ethanol diffusing into the permeate for our membranes, which is in the range of 55–65 wt% of permeate (Figure 6) compared, for example, with 10–20% reported by Hua et al. for feeds with similar (85% ethanol) composition [31]. However, it is important to note that our feed had a higher content of ethanol (around 88% and 92%). As explained above, even relatively small increases in the proportion of ethanol in the feed resulted in significant reductions in the permeance of water through the membrane while enhancing the permeance of ethanol manifold. In actual industrial applications, the permeate produced by our crosslinked GO membranes can be recycled to the initial ethanol dehydration step, which concentrates the ethanol feed prior to the pervaporation.

## 4. Conclusions

We report the performance of films of GO crosslinked by two types of molecules with branched structures, humic acid-like substances derived from urban wastes and a synthetic hyperbranched polyol, supported on commercial polyethersulfone membranes, for dehydration of ethanol by cross-flow pervaporation. The membranes can be easily fabricated by a very simple crosslinking reaction in aqueous solution with no unwanted byproducts, followed by rod-coating. This process can easily be scaled up for industrial-scale production of large-area membranes. Our membranes show a significantly higher flux than those reported by others for GO-based membranes. For a feed comprising around 90% by weight of ethanol, the permeate had a composition of 35–45% of water at operating temperatures in the range of 40–70 °C. Nevertheless, owing to the high flux achieved, the total membrane surface required for a given throughput will be low. Thus, these membranes could be potentially employed in a hybrid separation process consisting of distillation and pervaporation, with the permeate being recycled to the distillation unit. In contrast, supported GO membranes crosslinked by a linear molecule, poly(ethylene glycol), failed altogether in the continuous cross-flow pervaporation set-up despite having shown comparable stability and separation performance in a pervaporation set-up.

## Figures and Tables

**Table 1:** Characteristics of the four types of supported graphene oxide (GO) membranes used in this study.

Type of Membrane	Crosslinker/GO (w/w)	Interlayer Spacing (Å)
GO	-	6.55
GO-HBPO	0.2	7.93
GO-HAL	0.4	6.21
GO-PEG	10	12.42
